# Attention is required for canonical brain signature of prediction error despite early encoding of the stimuli

**DOI:** 10.1371/journal.pbio.3001866

**Published:** 2023-06-20

**Authors:** Alie G. Male, Robert P. O’Shea

**Affiliations:** 1 Discipline of Psychology, College of Science, Health, Engineering and Education, Murdoch University, Perth, Australia; 2 Department of Psychiatry and Human Behavior, School of Medicine, University of California, Irvine, United States of America; 3 Wilhelm Wundt Institute for Psychology, University of Leipzig, Leipzig, Germany; University of Cambridge, UNITED KINGDOM

## Abstract

Prediction error is a basic component of predictive-coding theory of brain processing. According to the theory, each stage of brain processing of sensory information generates a model of the current sensory input; subsequent input is compared against the model and only if there is a mismatch, a prediction error, is further processing performed. Recently, Smout and colleagues found that a signature of prediction error, the visual (v) mismatch negativity (MMN), for a fundamental property of visual input—its orientation—was absent without endogenous attention on the stimuli. This is remarkable because the weight of evidence for MMNs from audition and vision is that they occur without endogenous attention. To resolve this discrepancy, we conducted an experiment addressing 2 alternative explanations for Smout and colleagues’ finding: that it was from a lack of reproducibility or that participants’ visual systems did not encode the stimuli when attention was on something else. We conducted a similar experiment to that of Smout and colleagues. We showed 21 participants sequences of identically oriented Gabor patches, *standards*, and, unpredictably, otherwise identical, Gabor patches differing in orientation by ±15°, ±30°, and ±60°, *deviants*. To test whether participants encoded the orientation of the standards, we varied the number of standards preceding a deviant, allowing us to search for a decrease in activity with the number of repetitions of standards—repetition suppression. We diverted participants’ attention from the oriented stimuli with a central, letter-detection task. We reproduced Smout and colleagues’ finding of no vMMN without endogenous attention, strengthening their finding. We found that our participants showed repetition suppression: They did encode the stimuli preattentively. We also found early processing of deviants. We discuss various explanations why the earlier processing did not extend into the vMMN time window, including low precision of prediction.

## Abstract 2

Building on a previous PLOS Biology manuscript, this update article provides new evidence that canonical brain signatures of prediction error are only generated in the presence of attention to the visual stimuli, despite the fact that early brain encoding of the stimuli can be observed.

Our senses are flooded by information. For example, the 4 million cones of the human retina encode a staggering 101,200,000 bits of information at any instant if their responses to light were binary (which they are not) [1]. Still, we do not experience a flood—only a stream—of information to which we pay attention or of which we become conscious [2]. What we see does not require such an impossible burden of encoding because it is highly redundant, allowing prediction of the state of one photoreceptor at any instant from its state the instant before and from the state of its neighbors. This seminal idea developed into predictive-coding theory, first of the retina [3] and then of the brain’s hierarchical sensory systems [4] and ultimately to the whole brain [5–11].

Predictive-coding theory is a leading theory of how the brain deals with sensory input [12]. Friston [7,13–15] described the theory based on energy conservation and free-energy principles: It is that the brain uses past, bottom-up, sensory information, along with top-down information (such as prior probabilities, expectations, and attention) to generate predictive models of sensory input at various levels of the sensory pathway, linked by feedforward and feedback connections. To ensure good predictions, precision of predictions from previous bottom-up and top-down influences and from the success of previous predictions affect whether, or the degree to which, the model is updated [16–19]. For example, a prediction error for a small irregularity after high-precision predictions should prompt updating of the model, whereas the same irregularity after low-precision predictions may not [16].

We studied the visual mismatch negativity (vMMN) as our signature of prediction error [20]. The vMMN is an event-related potential (ERP) component from electroencephalography. Stefanics and colleagues [20] have reviewed its hundreds of studies: It occurs when a rare, unpredicted, *deviant*, visual stimulus occurs after a sequence of identical, *standard*, visual stimuli in a so-called oddball sequence. It is greater ERP negativity from parieto-occipital (PO) recording sites for deviants than for standards, occurring between 150 ms and 300 ms after the onset of the deviant. It is supposed to occur without endogenous attention on the deviant property of the stimuli.

There are 2 measures of the vMMN [20]. One is the *classic vMMN*, calculated as the difference between the ERP to deviants minus that to standards. The classic vMMN involves comparison of physically different stimuli and includes the greater neural adaptation of the frequently repeated standards than of the occasionally repeated deviants. The other is the *genuine vMMN*, calculated as the difference between the ERP to deviants minus that to some control (usually the *equiprobable control* [21,22]) stimuli, physically identical to the deviants and with equal neural adaptation, hence excluding it [23]. It has been used for hundreds of human ERP and animal single-cell experiments for various modalities. We focus on the genuine vMMN.

Recently, Smout and colleagues [24] found no ERP evidence of the genuine vMMN without endogenous attention to unexpected changes in orientation. In their attended condition, they showed each participant displays consisting of an annular Gabor patch (gratings of blurry dark and light bars) with a central gray patch containing a concentric black spot. They asked each participant to look only at the spot but to pay attention to the bars and to press a button on rare occasions when their spatial frequency increased. In their unattended condition, the same participant viewed the same displays but paid attention to the spot and pressed a button on rare occasions when its contrast decreased. The difficulty of the 2 tasks was equated for each participant in prior psychophysical testing.

Smout and colleagues’ stimuli of interest were the Gabor annuli. Participants viewed different-length sequences of displays of identical annuli—standards—followed by an otherwise identical annulus but with bars differing in orientation by ±20°, ±40°, ±60°, or ±80°—a deviant. On the next display, the deviant annulus was repeated, commencing a new sequence for which it now served as the standard—the so-called *roving-standard paradigm* [25]. To measure the genuine vMMN, Smout and colleagues used the equiprobable control.

Smout and colleagues found a genuine vMMN from PO electrodes to unexpected changes in the orientation of the bars in the attended condition (between about 170 ms to about 300 ms), but not in the unattended condition (their S1B Fig). They did find genuine, deviant-related responses in the unattended condition: a negativity from about 330 to 430 ms from a midline central electrode (Cz) and from about 300 to 470 ms from a midline frontal electrode (Fz). But these are too late to be considered vMMNs, or any sort of prediction error from such an early brain-processing feature of visual input as orientation [26].

Smout and colleagues confined their remaining analyses to their attended condition to answer questions about the timing and orientation tuning of prediction error. But the absence of a genuine vMMN without endogenous attention was surprising. Smout and colleagues did not discuss it except to cite one study for a similar finding in the auditory modality [27]. To resolve this discrepancy, we conducted an experiment addressing 2 alternative explanations for Smout and colleagues’ finding: that it was from a lack of reproducibility or that participants’ visual systems did not encode the stimuli when their endogenous attention was on something else.

We made some changes to improve our study’s ability to explore these alternatives. Smout and colleagues’ stimuli were Gabor annuli with an outer diameter of 11° diameter and an inner diameter of 0.83°. Following Smout and colleagues’ argument that bigger is better, we doubled the size of the outer diameter of our Gabor patches. Smout and colleagues’ inner gray patch interrupted the central bars over a region of about the size of the human foveola to which about 20% of the processing of the visual system is devoted [28]. To include as much as possible of that area, we eliminated any central gray patch, making our central bars continuous except for a very small area occupied by the lines of small task-relevant fixation letters.

We also omitted an attended condition, eliminating any possible confound in Smout and colleagues’ design from participants’ moving their eyes to the bars [29], thus bringing them onto central vision. We also analyzed clusters of electrodes around the single electrodes Smout and colleagues reported. This was to avoid issues related to variability in placement of single electrodes [30].

To look for evidence that participants encoded the orientation of the Gabor patches, we searched for repetition suppression: attenuated brain activity to repeated standard stimuli [31,32]. For Gabor patches similar to those used by Smout and colleagues and by us, this attenuation appears in various components of ERPs, as late as the P2 [32] and as early as the P1 at PO electrodes [33].

We found no genuine vMMN to unattended orientation deviants even though participants encoded the stimuli.

## Results

**Fig 1:**
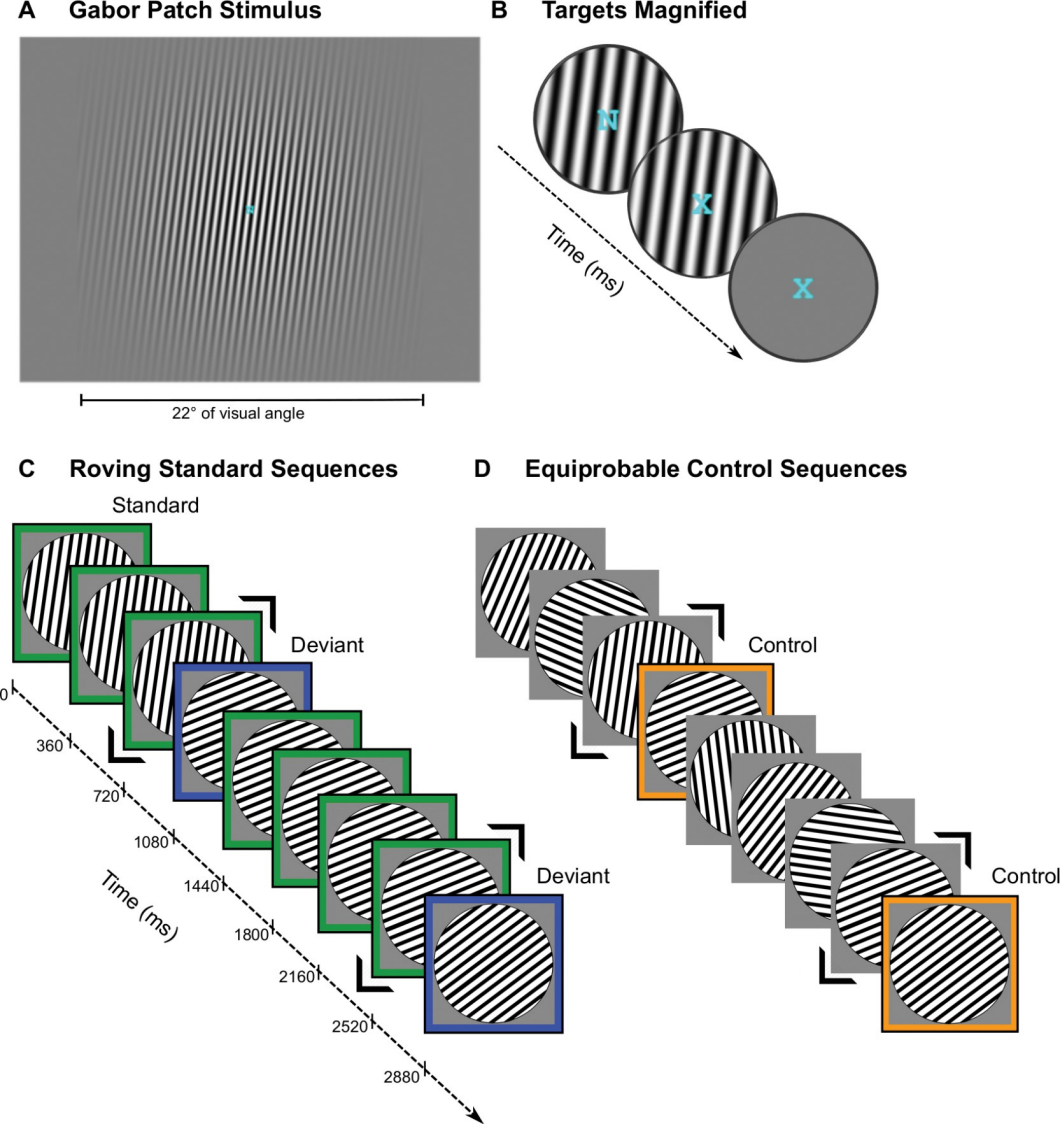
Experimental paradigm. **(A) Screenshots from the experiment of a Gabor patch** tilted 8° clockwise from vertical (0°), with a superimposed fixation letter. (**B) Magnified views of a sequence of target letters** superimposed on the Gabor patch (first 2 images) or on the gray screen during the interstimulus interval. The participants’ task was to press a key whenever an X appeared. Each letter was shown for 600 ms. (**C) Illustration of roving standard sequences** in which the first few trials of one sequence are 8°… 8°… 8°… *68*° (i.e., +60° orientation deviant for the first sequence), then…68°… 68°… 68°… 68°… *53*°… (i.e., −15° orientation deviant for the second sequence). Standards are outlined in green; deviants are outlined in blue. The stimulus preceding a deviant is framed with 2 black chevrons. (**D) Illustration of matching equiprobable controls** in which we replaced standard stimuli—except for the ones immediately preceding a deviant from the corresponding roving standard sequence—with Gabor patches whose orientation was randomly chosen among the 12 possible orientations. All orientations appeared equally (8.3%) often. The stimulus in the sequence (also framed with 2 black chevrons) preceding the one that would have been the deviant in the corresponding roving standard sequence had an identical orientation to that from the corresponding roving standard sequence. The control (outlined in orange) also had the same orientation as that of the deviant from the corresponding roving standard sequence. Gabor patch onset is indicated on the timeline. Stimuli appeared for 80 ms. The interstimulus interval was 280 ms.

Participants looked at a central, desynchronized stream of letters and pressed a key whenever an X appeared (Fig 1A and 1B). Desynchronized from the letter stream, Gabor patches appeared with, in roving-standard blocks, occasional changes in orientation after varying numbers of repetitions of the same orientation and, in equiprobable-control blocks, randomly selected orientations on each trial (Fig 1C and 1D).

### Did our participants attend to the letter-detection task, thereby ignoring the Gabor patches?

We checked whether our participants attended to the central, letter-detection task, thereby ignoring the Gabor patches. The mean hit rate for detecting the target X in the central letter stream was 98.22% and the mean false alarm rate was <0.1%, showing that participants paid attention to the task and did very well on it. There were no significant differences in performance of the task on roving-standard and equiprobable-control blocks (details of analyses in S 1 Supporting Information—Behavioral Results; S1 Data).

### Did we find a vMMN to unpredicted orientation changes?

**Fig 2:**
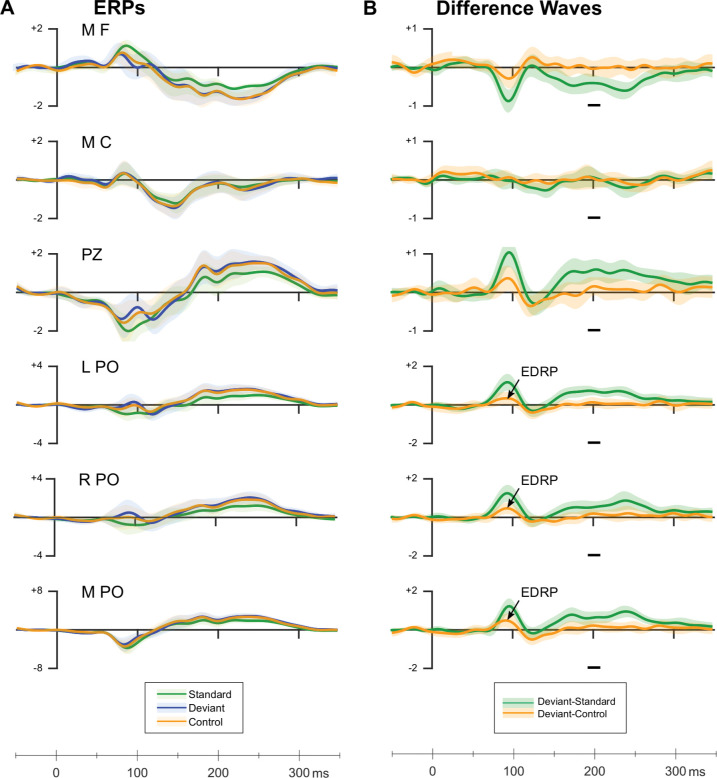
(A) **Grand average ERPs from various electrode clusters** around the electrodes Smout and colleagues reported. (B) **Difference waves for classic (60-degree deviant minus standard) and genuine (60-degree deviant minus 60-degree control) deviant-related activity**. The arrowed components show the only genuine EDRP. Horizontal gray bars illustrate the time window in which Kimura and Takeda [39] found the largest deviant-minus-control difference (i.e., genuine vMMN) for 32.7° orientation deviants. Mean amplitudes from this time window were analyzed using Bayesian replication tests (results in S3 Table). The lighter colors surrounding the difference waves give ±1 standard error of the mean. We used the same y-axis scales as Smout and colleagues [24] (data in S3 Data). EDRP, early deviant-related positivity; ERP, event-related potential; vMMN, visual mismatch negativity.

We did not find a genuine vMMN to unpredicted, unattended orientation changes. There is no hint of any genuine negativity in the vMMN time range to any unexpected orientation changes from any electrode cluster (we give details of our analyses, including Bayesian statistical tests, in S3 Table; S2 Data). We show the ERPs and the difference waves in Fig 2 (data in S3 Data), formatted similarly to those of Smout and colleagues. For simplicity, we give our results only for the ±60° orientation change, 10° larger than the average orientation change Smout and colleagues used. We give the ERPs and the difference waves for our other 2 orientation changes, ±15° and ±30°, in S1 and S2 Figs. Those difference waves also show no evidence of the vMMN. Preliminary inspection of ERPs confirm no vMMN for deviants in any length sequence of standards, i.e., we replicated Smout and colleagues’ finding no vMMN to unpredicted unattended changes in orientation, strengthening their finding (details in S1 Supporting Information—vMMN). To take another approach to describing our data, we used temporal principal component analysis (PCA; [34]). We found no component that was consistent with a vMMN.

**Fig 3:**
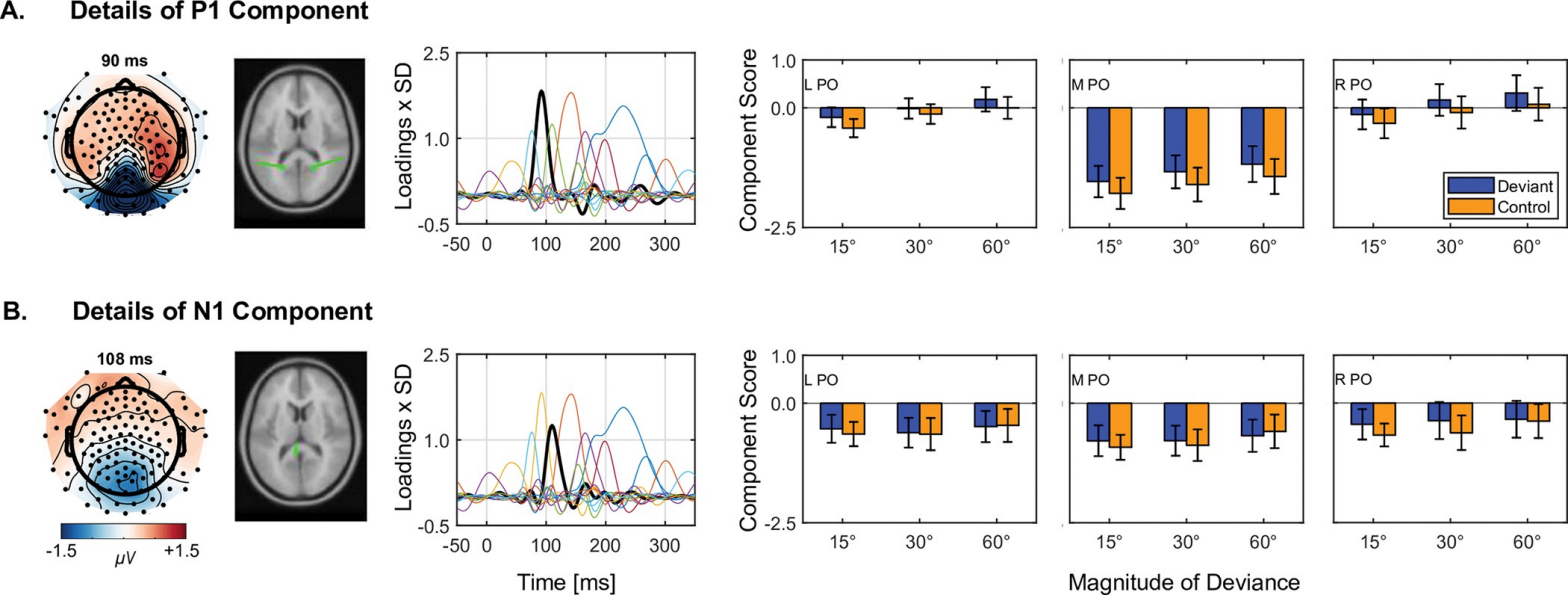
PCA component details for the P1 and N1 components of genuine deviant-related activity. **(A) PCA details of the P1.** (**B) PCA details of the N1**. The topographic maps in leftmost column show the combined activity from deviant and control trials at peak latency of the P1 (top) and N1 (bottom). In the second column, dipoles of each component help to show that the pronounced negativity at the M PO reflects the combined negative pole of both P1 and N1 components converging in close temporal and spatial proximity. In the third column, we show the component loadings (scaled by SD) (thick black line) relative to all other components (thin multicolored lines). This illustrates the component’s contribution to the overall evoked activity recorded from the scalp. In columns 4 to 6, we show the scores for deviant (orange) and control (purple) trials for each magnitude of deviance (15°, 30°, 60°) at the left (L), midline (M), and right PO region. Error bars depict ±1 standard error. Statistical analyses show that the deviant-related positivity at PO regions in Fig 2 are statistically significant for deviant-minus-control positivity for the P1 only (details in the Supporting information: S2.1; data in S4 Data). PCA, principal component analysis; PO, parieto-occipital.

We did find genuine, deviant-related activity outside the vMMN time range: a positivity we call the early deviant-related positivity (EDRP) from PO electrodes about 90 ms after onset of unpredicted orientation changes. PCA showed that this positivity was from the P1. Fig 3 illustrates these findings (details of analyses in S1 Supporting Information—Deviant-related positivity; data in S4 Data).

Smout and colleagues showed no sign of this early component in their unattended condition. This could be because they obscured the bars of the Gabor patch from central vision by their 0.83°-diameter gray patch, unlike in our study in which the bars crossed central vision.

### Did we find repetition suppression?

**Fig 4:**
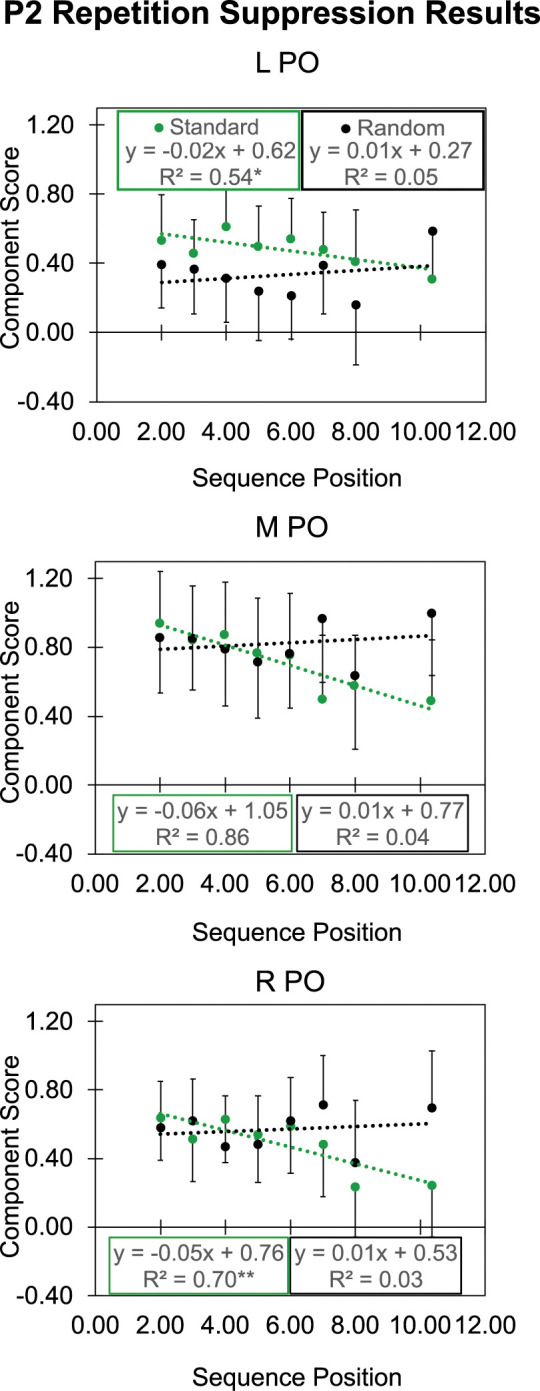
P2 repetition suppression results. P2 PCA scores are shown separately for the left (L), midline (M), and right (R) PO ROIs. Regression equations show the dotted lines in the data for standard and random stimuli. Asterisks denote the linear regression significance: *p < .05, **p < .01, ***p < .001. Error bars show 1 SE. We show +1 SE for standard and −1 SE for random stimuli to avoid overlap (data in S5 Data). PCA, principal component analysis; PO, parieto-occipital; ROI, region of interest; SE, standard error.

We did find repetition suppression. To search for it, we used PCA to identify plausible components and then tested their component scores against sequential position of preceding standards from 2 to ≥9. We needed to combine sequences showing ≥9 standards to have enough data for a reasonable average for that value. Its average was 10.36; we used that value in our regression model; it is the abscissa of that point in the graphs of Figs 4 and S3 (data in S5 Data). We found 2 components showing repetition suppression—the N1 with a peak latency of 94 ms (S3 Fig) and more clearly in the P2 with a peak latency of 268 ms (Fig 4).

Fig 4 shows that the PCA P2 scores became significantly less positive with increasing sequential position (i.e., repeated presentations) of standards (green points and regression lines). The P2 scores to random changes (black points and regression lines) became slightly more positive with sequential position, but not significantly. The results from the standard stimuli show clearly that our participants encoded them, so the absence of a vMMN cannot have been from lack of encoding.

## Discussion

The current study has 3 important findings:

- When unexpected, unattended changes to only the orientation of visual input occur, the typical brain signature of prediction error, the genuine vMMN, does not occur. This confirms the similar finding by Smout and colleagues, suggesting that theirs is not due to any reproducibility error.
- Participants encoded the stimuli by showing repetition suppression. The encoding extends into the time range of the vMMN, as others have reported [32,33,35,36].
- There is a sign of prediction error about 90 ms after onset of the stimuli: the EDRP.

Below, we consider why unattended, unexpected changes of orientation do not seem to produce a well-accepted neural signature of prediction error, the vMMN, and why they seem to produce an EDRP. We also consider the implications of our findings for existing models of attentional control and stimulus selection.

### Why no vMMN but an EDRP?

Absence of a genuine vMMN to unattended, unexpected changes in orientation is consistent with 5 experiments including ours [24,29,37,38], but inconsistent with 4 [22,39–41]. We consider 5 possible explanations:

1. There really is no genuine vMMN to unexpected, unattended orientation changes, and the 4 out of 9 studies that reported it are examples of publication bias or methodological errors.
2. There is a genuine vMMN to unexpected, unattended orientation changes, and the 5 studies that failed to find it suffered from some yet-to-be-determined methodological differences or problems.
3. Because the orientation changes were not attended, they were processed early, but not for long enough to engage the vMMN mechanism.
4. Endogenous attention is necessary for the vMMN.
5. The vMMN is affected by the EDRP.

### 1. No genuine vMMN to unexpected orientation changes?

A 44% hit rate across studies for finding a genuine vMMN to unexpected, unattended orientation changes is something to take notice of. But it is possible there are studies reporting no vMMN that never made it into print, which would reduce that rate. The vMMN literature does indeed show signs of publication bias. For example, of the 22 orientation-vMMN published studies, including those only on the classic vMMN, we are aware of only 4 reporting no vMMN for a hit rate of 82%. All but one [29] also reported a different condition or experiment in which a vMMN was found, suggesting that experiments in which all conditions failed to show a vMMN are either not submitted for publication or submitted and rejected. Such a bias would make it difficult to identify the experimental parameters required to resolve whether the genuine vMMN to orientation changes occur without attention.

### 2. Methodological differences or problems?

We considered whether our experimental parameters were responsible for our failure to find a genuine vMMN. We can rule out that our:

- Orientation differences (15° was our smallest) were too small, because observers can discriminate orientation differences between gratings of about 0.4° [42]. Moreover, the experiments that did find a genuine vMMN tested an orientation difference of 30° to 36° [22,39,43–45], whereas we and Smout and colleagues fruitlessly tested some much larger orientation differences.
- Presentation time (80 ms) was too short, because others have shown genuine vMMNs for other unattended, more complex deviants with presentation times as short as 17 ms [46].
- Our interstimulus interval (280 ms) was too short because others have shown genuine vMMNs with intervals as short as 210 ms [47].
- Our use of the roving-standard paradigm was responsible because others [24,48] did find a genuine orientation vMMN using it. Moreover, that paradigm has been used successfully to show MMNs to various deviants [9,49–51].

In a review of the literature, we noted that researchers generally failed to isolate individual low-level features, such as orientation, from other, confounding factors including eye movements that are capable of changing simple orientation changes into more complex visual changes [29]. Even Smout and colleagues’ study suffered from this, with very careful control of fixation in their unattended condition (the participants’ task was to detect contrast changes in a small central spot, requiring them to look at it) but not in their attended condition (the participants’ task was to detect spatial-frequency changes in the bars of the oriented stimuli, 0.42° away from the fixation spot). Male and colleagues [29] have shown evidence that participants’ eyes do stray away from central fixation when the task-relevant information is elsewhere, despite instructions to keep fixation central.

A potential limitation of our study is that we cannot discount the possibility that participants found the letter task so easy that endogenous attention, or even overt attention, drifted away from fixation. If so, we would anticipate some evidence of a vMMN given that Smout and colleagues found a mismatch signal when participants attended to the stimulus of interest. We did not find any such evidence. Additionally, if attention or distraction were different for roving-standard versus equiprobable-control sequences, we would anticipate differences in the ERP waveforms for deviant and control stimuli, if not in task performance; however, we found both to be markedly similar. We take this as a good indicator that our control condition, like Smout and colleagues’, was suitably designed for exploring unattended irregularities in low-level properties of visual input.

### 3. Unattended orientation changes are processed early, but not in the vMMN range?

Potentially, unattended orientation changes are processed early but not for long enough or for far enough in the visual pathways to engage the vMMN mechanism. This could explain our finding of the EDRP, our findings on repetition suppression, and our (and Smout and colleagues’) failure to find a vMMN to unattended stimuli. It would also be consistent with the theory that although unattended sensory inputs are fully preserved in the early, feedforward pathways for about 200 ms, only attended inputs are maintained for further processing in later, large-scale, recurrent networks for consciousness [52,53]. However, our and other researchers’ experimental data show that the repetition suppression we observed overlaps with the time range of the vMMN as does the processing of unattended inputs.

There is indeed experimental evidence that processing of unattended visual stimuli diminishes with time [54–56]. For example, Moerel and colleagues [56] showed participants superimposed rectangular gratings of different orientations and colors (blue and orange) for 100 ms; participants pressed a key when a cued grating (e.g., 90°, blue) appeared in a particular sequence of trials including all possible combinations of orientations and colors. They analyzed EEG data only from trials well before or after trials containing a cued or partially cued grating (e.g., matched the orientation but not the color). They trained pattern classifiers to discriminate 2 presented orientations 90° apart. Moerel and colleagues found that classifier accuracy was above chance for uncued orientations from about 80 ms after onset but diminished with time, whereas accuracy for cued orientations diminished less with time.

But the times for which unattended stimuli yield significant activity are all more than 400 ms after onset [54–56]. This is well into, and past, the vMMN range, from 150 ms to 300 ms. Even our P2, repetition-suppression result, with peak activity at 268 ms, is well beyond the notional time at which feedforward inputs are theoretically meant to give way to recurrent processing. We conclude that the processing of our unattended stimuli, both standards and deviants, endured well into the vMMN range.

### 4. Endogenous attention is necessary for the vMMN?

One could argue that we and Smout and colleagues did not find a vMMN because endogenous attention is necessary for it. Auksztulewicz and Friston [27] have made this proposition for auditory input. Doing so seems to us to undermine one of the most frequently cited evolutionary purposes of prediction error: to monitor one’s immense sensory input preattentively and to select only those that elicit prediction errors. We have no dispute with the notion that endogenous attention serves to increase the precision of prediction errors, as Smout and colleagues and others have argued [16,17,27], thereby engaging the vMMN mechanism. The neural mechanism is via regulation of the postsynaptic gain of error units undertaking the top down-sensory comparison and production of prediction errors.

### 5. The vMMN is affected by the EDRP?

We found evidence of an early prediction error, the genuine EDRP, at the P1, about 90 ms after onset of the Gabor patch. Of course, we need to be cautious about the EDRP’s reliability. Although we have found a similar early genuine, deviant-related positivity for orientation and for other low-level stimulus features [29] and others have reported early positivities for deviants either with a vMMN [57–60] and similar [61] or without [38], Smout and colleagues showed no sign of an EDRP. Theories on the early positivity range from memory-based change-detection, to changes in stimulus eccentricity [61], to hemi-field mediated deviance detection [60].

Nevertheless, we can think of 3 reasons why presence or absence of the vMMN might be affected by the EDRP:

- Energy efficiency,
- Stimulus complexity, and
- Precision of prediction.

#### Energy efficiency

One feature of predictive-coding theory is that it is designed for energy efficiency [7,14,15]. It is inefficient to respond to a prediction error at one level of the visual system if that error had been responded to at an earlier level. To decide whether this is reasonable, we had recourse to the better-studied auditory system.

When there are unexpected, unattended changes to auditory input, an MMN occurs from about 150 to 250 ms after onset [62–64]. More recently, ERP evidence has been found for processing of changes in physical properties of auditory input as early as 40 ms: the *middle-latency response* [65–70]. It might be akin to our EDRP.

However, one important characteristic of the middle-latency response is that it is always followed by the MMN, possibly because model updating is accomplished only by the MMN. This is consistent with predictive coding theory’s harmony with evolutionary reasons. A prediction error requires a listener to assess whether a preattentive stimulus represents a threat or a mating opportunity, thereby converting the stimulus into one demanding exogenous attention, yielding later ERP activity, in this case the MMN.

The visual EDRP does not show the same properties as the auditory middle-latency response: We found no hint of a vMMN after the EDRP. It will remain unknown whether the EDRP performs model updating until the EDRP is replicated and scrutinized for evidence of model updating. Although our study is not designed for revealing model updating, we hope that our findings will encourage studies better suited in pursuing the answer to this question.

#### Stimulus complexity

If the EDRP for simple features of visual input involves both detection of a prediction error and updating of the predictive model, it is possible that more complex aspects of visual input require the vMMN. Our Gabor patches changed only one low-level property—orientation—without any other low-level changes, such as of contrast, luminance, spatial frequency, or retinal location. Other studies with spatially complex figures, such as windmill patterns, providing more global, higher-level properties of the stimuli, have generally found a genuine vMMN [37,71–73].

#### Precision of prediction

The most likely tool in predictive-coding theory’s box is precision of prediction. This is the notion that variability of environment determines prediction precision, which, in turn, determines the prediction error [16,74]. Attention modulates precision [13,75]. Smout and colleagues found supporting evidence, admittedly only from their endogenously attended condition, by showing that the selectivity of a mismatch signal was greater when an unexpected orientation change followed more identical presentations of an unchanging orientation.

### Implications for models of attentional control and stimulus selection

There are various neural models of attentional control and stimulus selection [76–78]. They share being brain-wide neural pathways, focusing on separate but interacting pathways from the sensory cortices to higher levels: the *dorsal attention network* and the *ventral attention network* [79,80]. The dorsal network, involving goal-directed, endogenous attention, includes sensory cortices for whatever modality is required to accomplish a goal, such as the visual cortex when searching for red fruit in green foliage. It has feedforward and feedback connections to numerous brain areas mainly of the dorsal pathways including dorsal frontoparietal regions involved with attentional control. The dorsal network is active for as long as the searching continues, maintaining goal-directed behavior including finding and picking the fruit, enhancing processing in the visual cortex for features specific to red fruit, and suppressing the ventral network.

The ventral network also begins with the visual cortex when an unattended, unexpected change in visual input happens, such as the appearance of a venomous snake in the fruit tree. The visual cortex has other feedforward and feedback connections to numerous brain areas mainly of the ventral pathways including middle and inferior prefrontal regions. Then, both networks become transiently activated to accomplish the new goal-directed behavior: to escape from the snake.

Ideally, the activity of the 2 networks correspond to the components typically found from ERP studies of changes in visual input. When an unexpected change is unattended, the ventral network is supposed to mediate the vMMN [81]; when the unexpected change is attended (task relevant), then the dorsal network is supposed to mediate the later, P300 component [82]. But the coarse resolution of source localization from ERP studies casts some doubt on whether this ideal is true [79].

The speculative implication of our finding of the EDRP is that sometimes an unattended, unexpected orientation change can fail to activate the supposed ventral network’s vMMN. To distinguish this notion from some explanation via low precision of prediction will require studies beyond the scope of ours, perhaps with advanced source resolution or using dynamic causal modeling.

## Conclusion

We hope we have convinced you it is possible that unattended, unexpected changes in orientation do not yield the vMMN (consistent with the findings of Smout and colleagues) but might yield the EDRP. If so, the EDRP is a useful lead to understanding how the absence of a vMMN without attention could be reconciled with predictive-coding theory and models of attentional control and stimulus selection.

## Methods

We showed 21 [Smout and colleagues had 24] participants sequences of identically oriented Gabor patches (Fig 1A), standards, and, unpredictably, otherwise identical Gabor patches differing in orientation by ±15°, ±30°, and ±60° [Smout and colleagues used ±20°, ±40°, ±60°, and ±80°; we continue to give their values in brackets after ours], deviants (80 ms duration [100 ms], 280 ms [500 ms] interstimulus interval, 634 patches [415] per block; see Fig 1A). Participants looked at a central, desynchronized stream of letters and pressed a key whenever an X appeared (Fig 1B). Standard sequences ranged in length randomly from 3 to more than 11. For an equiprobable control, we presented the same stimuli, but in a random order (see Fig 1D).

### Ethics statement

The Human Research Ethics Committee at Murdoch University approved the experiment (permit 2015 208). All protocols adhered to the principles of the Declaration of Helsinki. All participants provided their written informed consent and were free to withdraw from the experiment at any time.

### Participants

Twenty-one self-declared healthy adults (8 males, 18 right-handed) with normal or corrected-to-normal vision participated in the experiment (statistical power = .88, based on Kimura and Takeda’s [39] reported effect size for genuine vMMN at PO8: Cohen’s *d* = −0.73). The mean age was 33 years with a range from 18 to 60 years. Most of the participants were undergraduate psychology students at Murdoch University. Participants received course credit or entry into a draw to win a $50 gift card in return for participation.

### Apparatus

Participants sat in a light-attenuated chamber viewing a calibrated monitor (17-inch, color, cathode ray tube display; Sony Trinitron Multiscan E230) from 57 cm. The monitor showed 1,280 × 1,024 pixels (75 Hz refresh rate) and was the only source of light. A chin rest stabilized each participant’s head. Participants gave their responses by pressing a key on a 4-key EGI response box with the index finger of their dominant hand.

A PC running Linux (v4.13.0), GNU Ubuntu (v16.04.4), Octave (v4.0.0) [83], and Psychophysics Toolbox (v3.0.14) [84–86] delivered the visual stimuli and recorded behavioral responses. An iMac running EGI’s NetStation 5.2 recorded EEG data.

### Stimuli

We used achromatic Gabor patches (mean RGB values of 128, 127, 128) on an average gray background (RGB values of 128, 127, 128). A Gabor patch comprises a grating of a particular spatial frequency and orientation whose contrast reduces with distance from the center of the grating according to a Gaussian function [87,88]. These stimuli are like those used by Smout and colleagues.

Gabor patches had a contrast of .999 [Smout and colleagues used 1.00], a phase of 0 radians—the black-to-white crossing was in the center of the patch [not specified], a spatial frequency of 2.4 cycles per degree of visual angle [2.73], and a standard deviation of the Gaussian of 3.84° of visual angle [not specified]. The visible parts of each Gabor patch had a diameter of approximately 22° of visual angle [11°, with a 0.83° central blank area]. There were 12 possible orientations: 8°, 23°, 38°, 53°, 68°, 83°, 98°, 113°, 128°, 143°, 158°, and 173° clockwise from vertical (0°) [9 possible orientations from 0° to 160°, in 20° steps] (further details in S1 Table).

For the participants’ primary task, capitalized letters were superimposed on the center of the Gabor patch in cyan (RGB values of 0, 255, 255) in 30-point Courier font [a black fixation spot]. Letters occupied 0.5° (width) × 0.6° (height) with a line width of 0.06° [visual angle of 0.3° diameter]. We used all letters of the English alphabet except for “I”. We give an illustration in Fig 1.

### Procedure

The participants’ task was to fixate on, and attend to, the continually changing random sequence of letters in the center of the monitor and to press a key with the dominant hand whenever an X appeared. Each letter occurred for 600 ms. None of the letters was immediately repeated. If the participant responded between 0.15 and 1.2 seconds after target onset, the response was correct. There were 383 letter changes during a block. On average, there were 15 targets (ranging from 7 to 25) per block.

Desynchronized from the letters, we presented our stimuli of interest—concentric Gabor patches—for 80 ms separated by a 280-ms interstimulus interval. We had 2 sorts of blocks.

### Roving-standard blocks

The experimental, roving-standard blocks contained 634 trials [Smout and colleagues: 415] showing Gabor patches of which 114 (17.98%) [59 (14.29%)] were deviants. Standard trials showed orientations from among twelve 15° steps from 8° clockwise from vertical (0°) [nine 20° steps from 0°]. Deviant Gabor patches differed in orientation from the preceding standard stimuli by ±15°, ±30°, or ±60° [±20°, ±40°, ±60°, or ±80°].

We used a hazard distribution to equalize the probability of the appearance of a deviant on any trial. Consequently, the distribution of numbers of standard trials against serial position had a gamma shape, with fewest trials for the largest serial positions [89,90]. We give the mean percentages of the numbers of standard-trial runs across all participants in S1 Table. On average, 5.53 standards preceded each deviant. Orientation for each standard was approximately 8.3% each. We give the mean numbers over participants in S1 Table.

### Equiprobable-control blocks

For each participant, we built equiprobable-control blocks from that participant’s roving-standard blocks. We replaced each block’s standard stimuli—except for the ones immediately preceding a deviant and the deviant itself—with a Gabor patch whose orientation was randomly chosen among the 12 possible orientations. The stimulus preceding the deviant and the deviant itself were identical in the 2 sorts of blocks. *Control trials* were the trials in the equiprobable-control block that were identical to the roving-standard block’s deviant trials. These 2 identical trials in both sorts of blocks allow us to discount the possibility that a difference in pairing of trials contributed to any deviant-related differences.

There were 6 roving-standard blocks and 6 equiprobable-control blocks. We randomized block order afresh for each participant. Each block took less than 4 minutes to complete. Participants were free to take breaks between blocks.

### EEG recording and analysis

We recorded the electroencephalogram (EEG) using an EGI, 129-channel, dense-array HydroCel geodesic sensor net, and Net Amps 300 signal amplifier. We recorded EEG at a 500-Hz sampling rate. Impedances were below 50 kΩ, per recommendation [91] and existing standard (e.g., [72,92–95]) for high-input impedance amplifiers. All electrodes were referenced to Cz.

We processed the EEG data offline using MATLAB 2015b (MathWorks, USA), EEGLAB 14.1.1 [96], and ERPLAB 6.1.4 [97]. We re-referenced the signal of all electrodes to the common average and filtered the EEG with a low-pass 40-Hz Kaiser-windowed (beta 5.65) sinc finite impulse response (FIR) filter (order 184) followed by a high-pass 0.1-Hz Kaiser-windowed (beta 5.65) sinc FIR filter (order 9056). Epochs were 400 ms long, featuring a 50-ms prestimulus period for baseline correction to accommodate the short 360 ms stimulus-onset-asynchrony. We excluded epochs with amplitude changes exceeding 800 μV at any electrode [98,99].

We identified electrodes with unusually high deviations in EEG activity relative to the average standard deviation pooled from all electrodes using the method described by Bigdely-Shamlo and colleagues [100]. A robust z-score was calculated for each electrode by replacing the mean by the median and the standard deviation by the robust standard deviation (0.7413 times the interquartile range). We removed any electrode with a robust z-score exceeding 2.0 provided at least 4 others surrounded it for later interpolation.

We created vertical and horizontal EOG channels by bipolarizing data from electrodes above and below the right eye (electrodes 8 and 126) and outer canthi of both eyes (electrodes 1 and 32), respectively (as in [72]). To ensure that trials where participants moved their eyes or blinked were not included in the final analysis of the data, we identified epochs containing amplitude changes exceeding ±60 μV at these EOG channels for rejection prior to ICA correction. We did not exclude these epochs at this stage, and ICA artifact correction had not yet occurred.

We performed independent component analysis (ICA) with AMICA [101]. To improve the decomposition, we performed the analysis on raw data (excluding bad electrodes) that was first filtered using a 1-Hz high-pass (Kaiser-windowed sinc FIR filter, order 804, beta 5.65) and 40-Hz low-pass filter as suggested by Winkler and colleagues [98]. Thereafter, we segmented into epochs, but without baseline correction [102]. We simultaneously reduced the data to 32 components.

We applied the demixing matrix to the 0.1- to 40-Hz filtered data and used SASICA [103] to identify which components exhibited low autocorrelation, low focal electrode or trial activity, high correlation with vertical or horizontal EOG, or met ADJUST criteria [104]. We assessed the remaining components using criteria described by Chaumon and colleagues [105], classifying components based on consistent activity time-locked to stimulus onset across all trials, on topography, or on power spectrum. We removed components identified as unrelated to brain activity.

We then removed epochs that were previously identified for rejection. This was followed by a final artifact rejection—removing epochs containing amplitude changes exceeding ±60 μV at any electrode. Finally, using spherical splines [106], we interpolated data for removed electrodes.

For exploring deviant-related responses, we averaged ERPs for the standard, deviant, and control trials, excluding epochs immediately following a deviant or control trial. We produced difference waves by subtracting ERPs to standards and ERPs to controls from ERPs to deviants.

For exploring repetition suppression, we averaged PCA components for standards from roving-standard blocks and for randomly oriented stimuli from equiprobable-control blocks at varying sequential positions, excluding deviant and control trials. We retained standard trials immediately following deviants; we retained randomly oriented trials immediately following controls (S1 Table for number of epochs in each ERP).

We defined anterior, central, and PO regions of interest (ROIs). The signal from all electrodes within each ROI was averaged over time. We visually inspected ERPs for electrodes within each ROI and then when averaged together. There was middle (M) frontal (M F) central (M C), parieto-occipital (M PO), left (L) frontal (L F), central (M C), parieto-occipital (L PO), and right frontal (R F), central (R C), and parieto-occipital (R PO). We display the results of 6 ROIs (M F, M C, M PO, L PO, R PO; see S2 Table for electrodes within each cluster) and 1 PZ electrode for comparison with Smout and colleagues.

Using the EP Toolkit (v2.64; [34]), we conducted temporal PCA on the individual average ERP data from all 129 electrodes, thereby allowing for deviant-related responses isolated to any one electrode to emerge. For exploring deviant-related responses, we included deviant and control trials. For exploring repetition suppression, we included standard and random trials. We used Promax orthogonal rotation (κ = 3) with a covariance relationship matrix and Kaiser weighting as recommended by Dien and colleagues [107]. PCA reduced the data to those components explaining most of the observed data. The component that explained the greatest amount of signal is component 1, and every component thereafter explained less of the data than the component before it. We show how much of the signal a component is responsible for by plotting the component’s loading (scaled by SD) over time [108].

Using PCA, one can identify separate components in the ERP waveform and extract an alternative measure of ERP component amplitudes for inferential testing [107,109,110]. Each PCA component has a peak latency, a site of maximum positivity on the scalp (i.e., a component’s positive pole), and a site of maximum negativity on the scalp (i.e., a component’s negative pole). Plotting a topographical map of the microvolt scaled PCA data of a single component at the time of its peak latency shows the component’s positive and negative pole.

A vMMN component would emerge as a component that is largest (most negative) between 150 and 300 ms, based on previous vMMN studies using orientation deviants (e.g., 162 to 170 ms in [43]; 200 to 250 ms in [22]; 190 to 220 ms in [39]). A genuine vMMN component should also yield scores that are more negative for deviants compared to controls at the component’s negative pole.

In addition to our frequentist paired *t* tests, we conducted Bayesian paired *t* tests to determine the likelihood of obtaining the data. We used a medium prior (with a Cauchy prior whose width was set to 0.707) for all Bayesian analyses. For interpreting our findings, a model with the largest Bayes Factor (*BF*_10_) is the model that best explains the data; this is the favored model. All main effects and interactions in the favored model are, therefore, important for explaining the data. Evidence against the null is considered weak if a *BF*_10_ is between 1 and 3. It is positive for a *BF*_10_ between 3 and 20, strong for a *BF*_10_ between 20 and 150, and very strong given a *BF*_10_ greater than 150 [111].

We also performed Bayes Factor replication (*BF*_r0_) tests [112] on mean amplitudes between 197 and 207 ms at each PO ROI given a prior reflecting the effect size (Cohen’s *d* = −0.73) for the genuine vMMN at the PO8 in Kimura and Takeda’s [39] oddball condition for 32.7° orientation deviants.

For all frequentist ANOVAs, we applied the Greenhouse–Geisser correction where necessary (ε < .750). Eta squared (η^2^) denotes the estimated effect size.

## Supporting information

S1 Fig (A) **Grand average ERPs for 15° orientation changes from various electrode clusters** around the electrodes Smout and colleagues [24] reported. (B) **Difference waves for classic (15-degree deviant minus standard) and genuine (15-degree deviant minus control) deviant-related activity**. The arrowed components show the only genuine EDRP. Horizontal gray bars illustrate the time-window in which Kimura and Takeda [39] found the largest deviant-minus-control difference (i.e., genuine vMMN) for 32.7° orientation deviants. Mean amplitudes from this time window were analyzed using Bayesian replication (results in S3 Table). The lighter colors surrounding the difference waves give ±1 standard error of the mean (data in S3 Data). EDRP, early deviant-related positivity; ERP, event-related potential; vMMN, visual mismatch negativity.
(PDF)

S2 Fig (A) **Grand average ERPs for 30° orientation changes from various electrode clusters** around the electrodes Smout and colleagues [24] reported. (B) **Difference waves for classic (30-degree deviant minus standard) and genuine (30-degree deviant minus control) deviant-related activity**. The arrowed components show the only genuine EDRP. Horizontal gray bars illustrate the time-window in which Kimura and Takeda [39] found the largest deviant-minus-control difference (i.e., genuine vMMN) for 32.7° orientation deviants. Mean amplitudes from this time window were analyzed using Bayesian replication (results in S3 Table). The lighter colors surrounding the difference waves give ±1 standard error of the mean (data in S3 Data). EDRP, early deviant-related positivity; ERP, event-related potential; vMMN, visual mismatch negativity.
(PDF)

S3 Fig N1 repetition suppression results. N1 PCA scores are shown separately for the left (L), midline (M), and right (R) PO ROIs. Regression equations show the dotted lines in the data for standard and random stimuli. Asterisks denote the linear regression significance: *p < .05, **p < .01, ***p < .001. Error bars show 1 SE. We show −1 SE for standard and +1 SE for random stimuli to avoid overlap (data in S5 Data). PCA, principal component analysis; PO, parieto-occipital; ROI, region of interest; SE, standard error.
(PDF)

S1 Data The number of trials, targets, targets correctly identified (Hit N), targets not identified (Miss N), targets incorrectly identified (False Alarm N), mean reaction time (RT), hit rate, and false alarm rate for Roving and Equiprobable sequences appear for each participant on separate rows. Means and standard deviations for Roving and Equiprobable sequences appear in the last 2 rows. Data summary and analysis results in S1 Supporting Information—Behavioral Results.
(XLSX)

S2 Data Mean amplitudes and difference wave mean amplitudes (μv) at left (L), middle (M), and right (R) parieto-occipital (PO) regions between 197 and 207 ms for each magnitude of deviance (Small 15-degree Deviant, Medium 30-degree Deviant, Large 60-degree Deviant) and condition (Standards, Deviants, Controls, Deviant-minus-Standard, and Deviant-minus-Control) for each participant on separate rows. Data summary and analysis results in S3 Table.
(XLSX)

S3 Data Amplitudes (μv) over time (−50 to 348 ms) at middle (M) frontal (M F), middle central (M C), middle parieto-occipital (M PO), left parieto-occipital (L PO), right parieto-occipital (R PO), and parietal (PZ) regions for each magnitude of deviance (Small 15-degree Deviant [S1 Fig], Medium 30-degree Deviant [S2 Fig], Large 60-degree Deviant [Fig 2]) and condition (Standards, Deviants, Controls, Deviant-minus-Standard, and Deviant-minus-Control) for each participant on separate rows. (XLSX)

S4 Data P1 and N1 principal component scores at left parieto-occipital (L PO), middle parieto-occipital (M PO), and right parieto-occipital (R PO) regions for each magnitude of deviance (Small 15-degree Deviant, Medium 30-degree Deviant, Large 60-degree Deviant) and condition (Standards, Deviants, Controls, Deviant-minus-Standard, and Deviant-minus-Control) for each participant on separate rows. Data summary and analysis results in S1 Supporting Information—Deviant-related positivity and Fig 3.
(XLSX)

S5 Data Means and standard errors for P2 and N1 principal component scores at left parieto-occipital (L PO), middle parieto-occipital (M PO), and right parieto-occipital (R PO) regions for standard and random stimuli at positions: 2, 3, 4, 5, 6, 7, 8, and 10.36 (the average number of ≥9 standards). Data summary and analysis results in S1 Supporting Information—Repetition suppression and Figs 4 (P2) and S3 (N1).
(XLSX)

S1 Table Orientation probabilities for standard and random stimuli appearing in roving-standard and equiprobable-control blocks, the number of standard repetitions, and the mean number of epochs (and standard deviations) for each event-related potential (ERP). (DOCX)

S2 Table Electrode numbers on 128-channel geodesic sensor net within left, middle, right, frontal, parieto-occipital, and central regions of interest (ROIs) with corresponding 10–20 electrodes. (DOCX)

S3 Table Statistical analysis of the difference wave mean amplitudes at the left, middle, and right parieto-occipital regions of interest (ROIs) between 197 and 207 ms for each magnitude of deviance (Small 15-degree Deviant, Medium 30-degree Deviant, Large 60-degree Deviant). (DOCX)

S1 Supporting Information Results of statistical analysis of behavioral (S1) and electrophysiological (S2) data presented in the main text. Behavioral results include hit rates, false alarm rates, and reaction times. Electrophysiological data include deviant-related responses and repetition suppression.
(DOCX)

## Abbreviations

EDRP: early deviant-related positivity
EEG: electroencephalogram
ERP: event-related potential
FIR: finite impulse response
ICA: independent component analysis
MMN: mismatch negativity
PCA: principal component analysis
PO: parieto-occipital
ROI: region of interest
vMMN: visual mismatch negativity
