# Supplementary figures and images for: Attention is required for canonical brain signature of prediction error despite early encoding of the stimuli
Source: PLoS Biol. 2023 Jun 20;21(6):e3001866. doi: 10.1371/journal.pbio.3001866 (PMC10281583; doi:10.1371/journal.pbio.3001866)

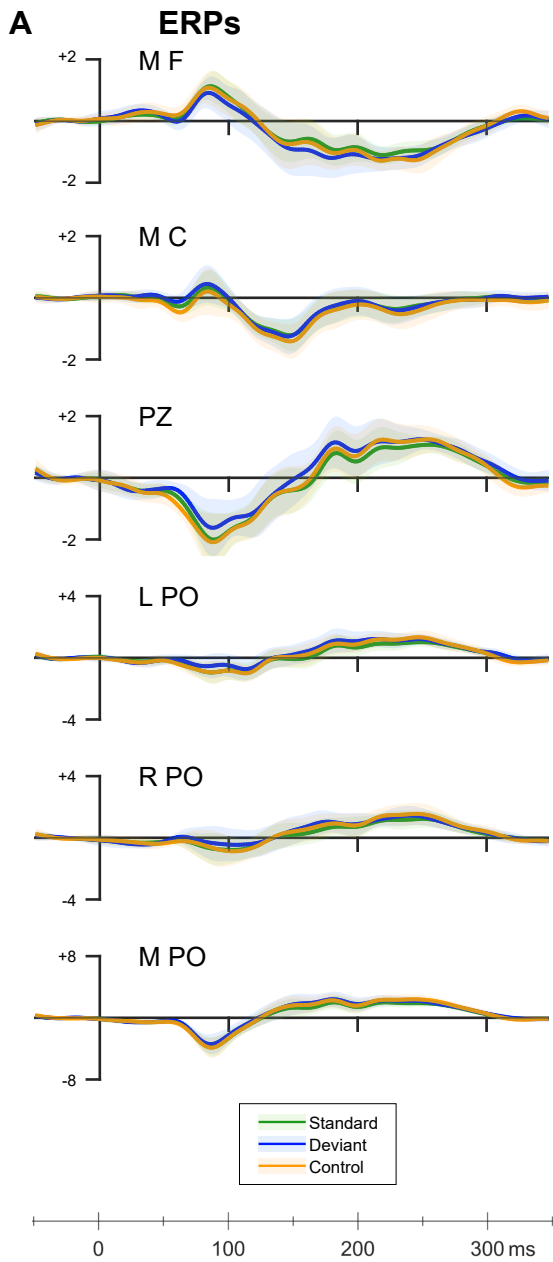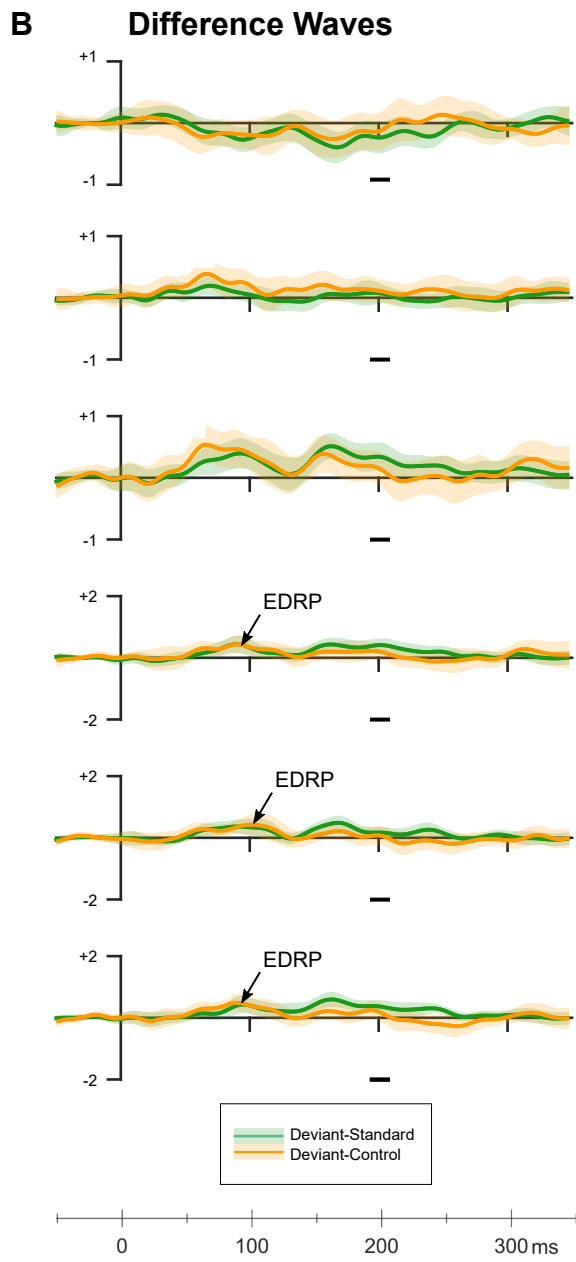

Supplement: S1 Fig — (A) Grand average ERPs for 15° orientation changes from various electrode clusters around the electrodes Smout and colleagues [24] reported. (B) Difference waves for classic (15-degree deviant minus standard) and genuine (15-degree deviant minus control) deviant-related activity. The arrowed components show the only genuine EDRP. Horizontal gray bars illustrate the time-window in which Kimura and Takeda [39] found the largest deviant-minus-control difference (i.e., genuine vMMN) for 32.7° orientation deviants. Mean amplitudes from this time window were analyzed using Bayesian replication (results in S3 Table). The lighter colors surrounding the difference waves give ±1 standard error of the mean (data in S3 Data). EDRP, early deviant-related positivity; ERP, event-related potential; vMMN, visual mismatch negativity. (PDF) [file pbio.3001866.s001.pdf]

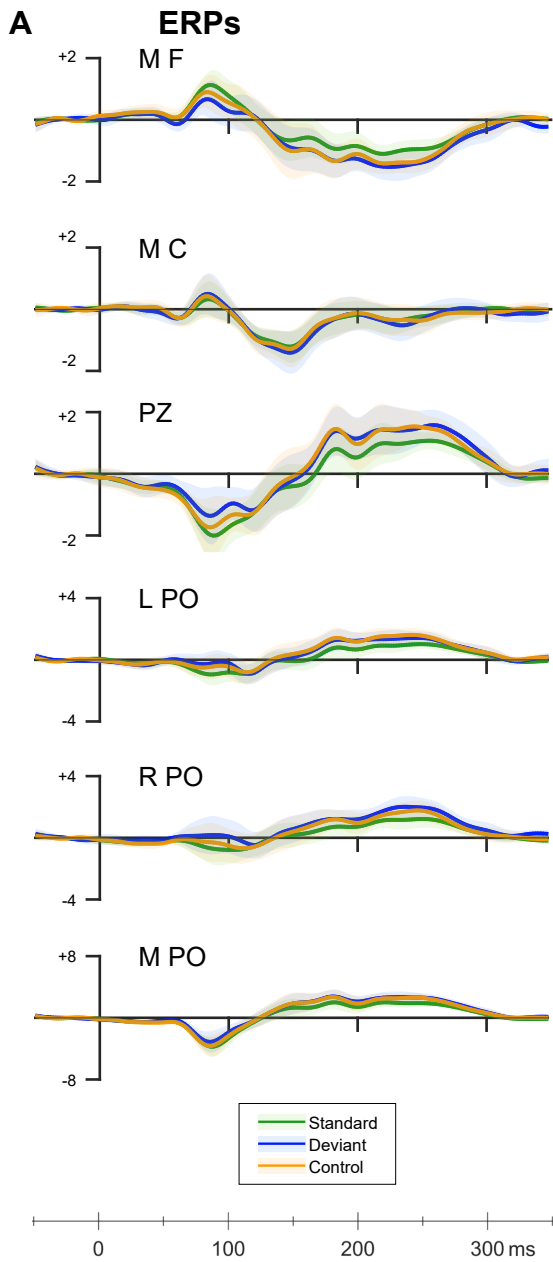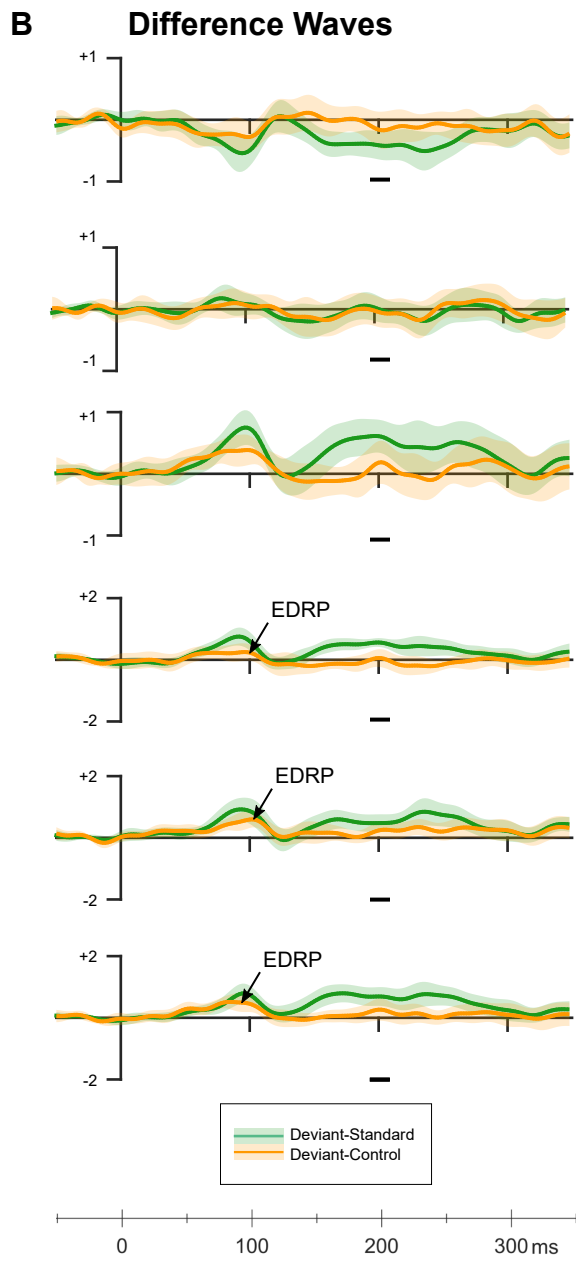

Supplement: S2 Fig — (A) Grand average ERPs for 30° orientation changes from various electrode clusters around the electrodes Smout and colleagues [24] reported. (B) Difference waves for classic (30-degree deviant minus standard) and genuine (30-degree deviant minus control) deviant-related activity. The arrowed components show the only genuine EDRP. Horizontal gray bars illustrate the time-window in which Kimura and Takeda [39] found the largest deviant-minus-control difference (i.e., genuine vMMN) for 32.7° orientation deviants. Mean amplitudes from this time window were analyzed using Bayesian replication (results in S3 Table). The lighter colors surrounding the difference waves give ±1 standard error of the mean (data in S3 Data). EDRP, early deviant-related positivity; ERP, event-related potential; vMMN, visual mismatch negativity. (PDF) [file pbio.3001866.s002.pdf]

# N1 Repetition Suppression Results

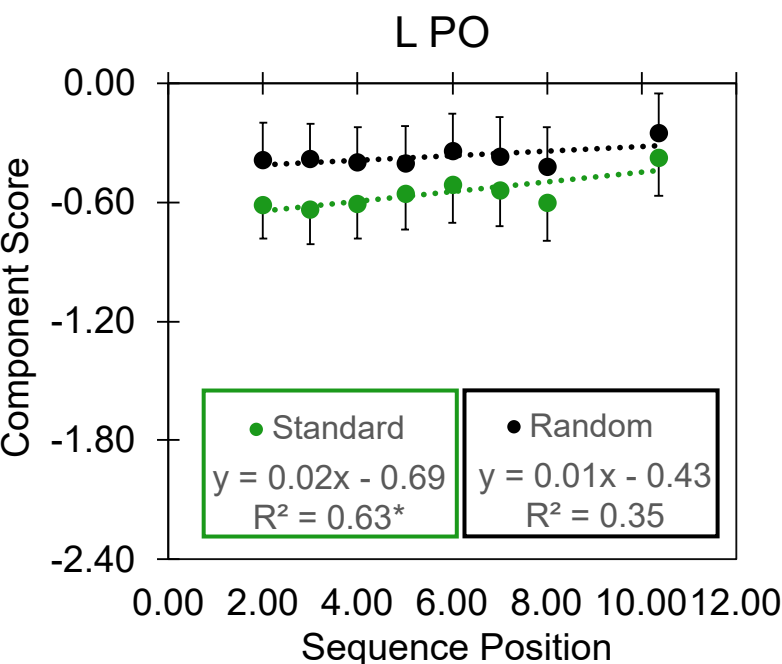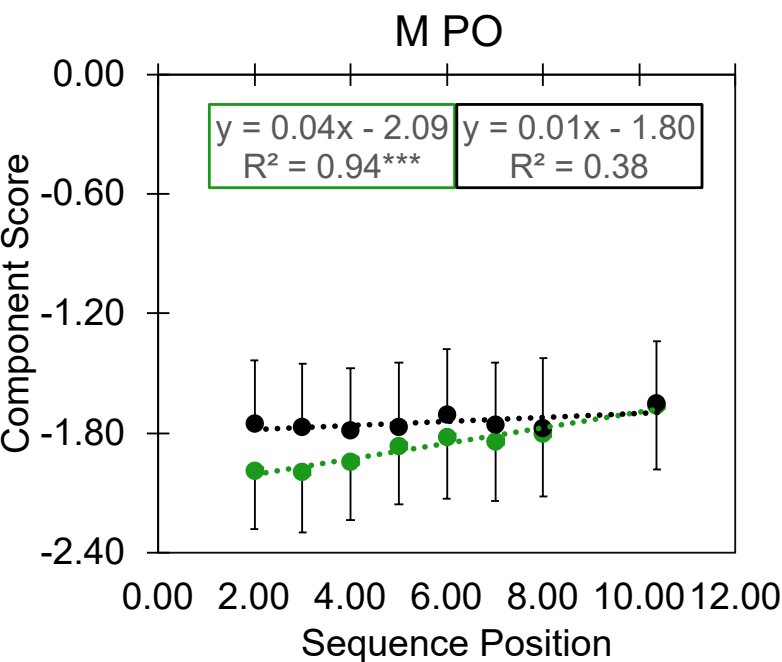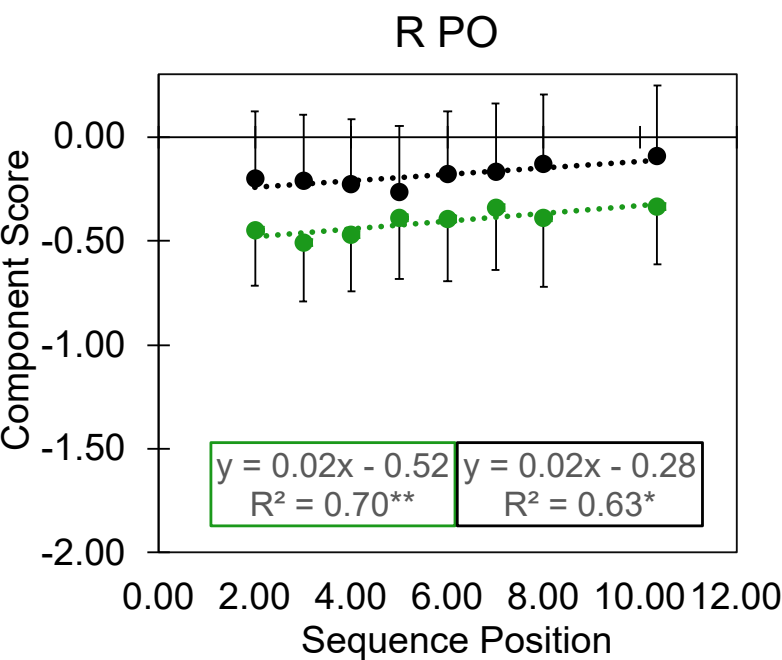

Supplement: S3 Fig — N1 PCA scores are shown separately for the left (L), midline (M), and right (R) PO ROIs. Regression equations show the dotted lines in the data for standard and random stimuli. Asterisks denote the linear regression significance: *p < .05, **p < .01, ***p < .001. Error bars show 1 SE. We show −1 SE for standard and +1 SE for random stimuli to avoid overlap (data in S5 Data). PCA, principal component analysis; PO, parieto-occipital; ROI, region of interest; SE, standard error. (PDF) [file pbio.3001866.s003.pdf]
