# Supplementary material for: Attention is required for canonical brain signature of prediction error despite early encoding of the stimuli
Source: PLoS Biol. 2023 Jun 20;21(6):e3001866. doi: 10.1371/journal.pbio.3001866 (PMC10281583; doi:10.1371/journal.pbio.3001866)
Supplement: S1 Table — (DOCX) [file pbio.3001866.s009.docx]

Supporting Materials – Tables

S1 Table

*Orientation Probabilities for Standard and Random Stimuli, Number of Standard Repetitions, and the Mean Number of Epochs (Standard Deviations) in each ERP*

| **Property** | **Stimulus Condition** | | |  |  |
| --- | --- | --- | --- | --- | --- |
| Orientation° (Probability %) | Standard | Random | |  |  |
| 8 | 8.55 | 8.3 | |  |  |
| 23 | 8.51 |  | |  |  |
| 38 | 7.89 |  | |  |  |
| 53 | 8.31 |  | |  |  |
| 68 | 8.05 |  | |  |  |
| 83 | 8.17 |  | |  |  |
| 98 | 8.47 |  | |  |  |
| 113 | 8.16 |  | |  |  |
| 128 | 8.64 |  | |  |  |
| 143 | 8.42 |  | |  |  |
| 158 | 8.36 |  | |  |  |
| 173 | 8.47 |  | |  |  |
|  |  | | Mean Number of Epochs (SD) | | |
| **Focus** | **Stimulus Condition** | | | | |
|  | Standard | Random | | Deviant | Control |
| Repetition Suppression |  |  | |  |  |
| Position (Probability %) |  |  | |  |  |
| 2 | 561 (76) | 563 (89) | |  |  |
| 3 (16.03) | 568 (73) | 570 (86) | |  |  |
| 4 (21.69) | 473 (65) | 478 (73) | |  |  |
| 5 (19.77) | 350 (47) | 351 (54) | |  |  |
| 6 (16.06) | 239 (35) | 239 (41) | |  |  |
| 7 (10.42) | 149 (23) | 147 (26) | |  |  |
| 8 (6.65) | 90 (12) | 90 (14) | |  |  |
| ≥ 9 (9.38) | 120 (21) | 123 (22) | |  |  |
| Deviant-Related Activity |  |  | |  |  |
| 15-degrees Deviant |  |  | | 187 (26) | 187 (28) |
| 30-degrees Deviant |  |  | | 186 (28) | 188 (30) |
| 60-degrees Deviant | 1994 (261) |  | | 188 (25) | 188 (27) |

*Note.* Minimum of three standards preceded each deviant. All standards combined for deviant-related activity analyses.
