# Supplementary material for: Attention is required for canonical brain signature of prediction error despite early encoding of the stimuli
Source: PLoS Biol. 2023 Jun 20;21(6):e3001866. doi: 10.1371/journal.pbio.3001866 (PMC10281583; doi:10.1371/journal.pbio.3001866)
Supplement: S2 Table — (DOCX) [file pbio.3001866.s010.docx]

Supporting Materials – Tables

S2 Table

*Electrode Numbers on 128-Channel Geodesic Sensor Net within Regions of Interest (ROI) with Corresponding 10-20 Electrodes*

|  | **ROI Abbreviation** | **Number of Electrodes** | **EGI Electrode Numbers** |
| --- | --- | --- | --- |
| Frontal |  |  |  |
| Left | L F | 4 | 24 [F3], 23, 27, 19 |
| Middle | M F | 2 | 11 [FZ], 16 |
| Right | R F | 4 | 124 [F4], 4, 3, 123 |
| Parieto-occipital |  |  |  |
| Left | L PO | 4 | 59, 60, 65, 66 |
| Middle | M PO | 2 | 75 [OZ], 72 |
| Right | R PO | 4 | 91, 84, 85, 90 |
| Central |  |  |  |
| Left | L C | 4 | 36 [C3], 30, 37, 42 |
| Middle | M C | 2 | Ref [CZ], 55 |
| Right | R C | 4 | 104 [C4], 93, 105, 87 |
| PZ | PZ | 1 | 62 |
