# Supplementary material for: Attention is required for canonical brain signature of prediction error despite early encoding of the stimuli
Source: PLoS Biol. 2023 Jun 20;21(6):e3001866. doi: 10.1371/journal.pbio.3001866 (PMC10281583; doi:10.1371/journal.pbio.3001866)
Supplement: S3 Table — (DOCX) [file pbio.3001866.s011.docx]

Supporting Materials – Tables

S3 Table

*Directed Paired (BF_10_) and Replication (BF_r0_) Bayesian t-tests of the Difference Wave Mean Amplitudes (µV) at Left (L), Middle (M), and Right (R) Parieto-occipital (PO) Regions Between 197 and 207 ms for Each Magnitude of Deviance (df = 20)*

|  | **Deviant vs. Standard (Classic)** | | | | | **Deviant vs. Control (Genuine)** | | | | |
| --- | --- | --- | --- | --- | --- | --- | --- | --- | --- | --- |
|  | **µV** | ***t*** | ***p*** | ***BF*_10_** | ***BF*_r0_** | **µV** | ***t*** | ***p*** | ***BF*_10_** | ***BF*_r0_** |
| *L PO* | | | | | | | | | | |
| 15° (small) | 0.41 | 3.879 | .999 | 0.061 | 0.009 | 0.18 | 1.516 | .927 | 0.101 | 0.012 |
| 30° (medium) | 0.55 | 7.701 | .999 | 0.016 | 0.014 | 0.04 | 0.294 | .614 | 0.185 | 0.038 |
| 60° (large) | 0.71 | 6.871 | .999 | 0.018 | 0.014 | 0.12 | 0.962 | .826 | 0.127 | 0.018 |
| *M PO* | | | | | | | | | | |
| 15° (small) | 0.36 | 3.471 | .998 | 0.064 | 0.008 | 0.16 | 1.060 | .849 | 0.122 | 0.017 |
| 30° (medium) | 0.69 | 4.597 | .999 | 0.056 | 0.009 | 0.25 | 1.465 | .921 | 0.103 | 0.012 |
| 60° (large) | 0.64 | 4.280 | .999 | 0.058 | 0.009 | 0.22 | 1.810 | .957 | 0.092 | 0.011 |
| *R PO* | | | | | | | | | | |
| 15° (small) | 0.17 | 1.933 | .966 | 0.088 | 0.010 | 0.06 | 0.387 | .649 | 0.174 | 0.033 |
| 30° (medium) | 0.48 | 3.464 | .999 | 0.064 | 0.008 | 0.25 | 1.892 | .963 | 0.089 | 0.010 |
| 60° (large) | 0.54 | 3.617 | .999 | 0.063 | 0.008 | 0.19 | 1.822 | .958 | 0.091 | 0.010 |

*Note*. Time-window is the same as the time-window in which Kimura and Takeda [1] found the largest deviant-minus-control difference at PO8 for orientation deviants of 32.7°. Supplementary materials S2 Data.

Reference

[1] Kimura M, Takeda Y. Automatic prediction regarding the next state of a visual object: Electrophysiological indicators of prediction match and mismatch. Brain Research. 2015:31-44. https://doi.org/10.1016/j.brainres.2015.01.013. PubMed PMID: 25598206.
