# Supplementary material for: Attention is required for canonical brain signature of prediction error despite early encoding of the stimuli
Source: PLoS Biol. 2023 Jun 20;21(6):e3001866. doi: 10.1371/journal.pbio.3001866 (PMC10281583; doi:10.1371/journal.pbio.3001866)
Supplement: S1 Supporting Information — Behavioral results include hit rates, false alarm rates, and reaction times. Electrophysiological data include deviant-related responses and repetition suppression. (DOCX) [file pbio.3001866.s012.docx]

**Supplementary Materials**

1. Behavioral Results

Hit rates for roving-standard blocks (*M*=98.22%, *SD*=1.84%) were essentially identical to those for equiprobable-control blocks (*M*=98.22%, *SD*=2.31%), *t*(20) =0.016, *p*=.987, *BF*_10_=0.228. False alarm rates were similar in roving-standard (*M*=0.07%, *SD*=0.07%) and equiprobable-control blocks (*M*=0.05%, *SD*=0.05%), *t*(20)=1.678, *p*=.109, *BF*_10_=0.754. Reaction times hazard were essentially identical in the roving-standard (*M*=545.30 ms, *SD*=51.89 ms) and equiprobable-control blocks (545.39 ms, *SD*=51.25 ms), *t*(20)=‑0.025, *p*=.981, *BF*_10_=0.228 (S1_Data).

1. Electrophysiological Results
   1. **vMMN**

Bayesian replication confirmed that there was neither a classic nor a genuine vMMN (complete analyses in S3 Table: S2_Data). In fact, for the classic vMMN (green traces), there was a positivity in the analyzed time window—the opposite of a vMMN. Smout et al. [1] showed the same positivity.

- 1. **Deviant-related positivity**

For the early deviant-related positivity (EDRP), we performed a 3 × 3 × 2 repeated-measures ANOVA on P1 and N1 scores with parietal-occipital (PO) region (left vs. middle vs. right), magnitude of deviance (15° vs. 30° vs. 60°), and stimulus type (deviant vs. control) as factors (S4_Data).

For P1, magnitude of deviance was significant, *F*(2, 40)=26.811, *p*< .001, η^2^=.011, ε=.667. Holm post-hoc tests showed significantly larger scores for 60° orientation change compared with 15° (*t*=‑7.282, *p*<.001, *d*=‑0.280) and with 30° (*t*=‑2.974, *p*=.005, *d*=‑0.114) and for 30° compared with 15° (*t*=‑4.308, *p*<.001, *d*=‑0.166). Smout et al. [1] found similar results, albeit in their attended condition.

A ROI × stimulus interaction emerged, *F*(2,40)=3.547, *p*=.038, η**^2^**<.001, ε**=**.907. Holm post-hoc tests showed deviants were significantly more positive than controls at LPO (*t*=2.882, *p*=.041, *d*=0.123), MPO (*t*=3.990, *p*=.003, *d*=4.293), and RPO (*t*=3.786, *p*=.010, *d*=3.786). The data provide very strong evidence for the favored Bayesian model including the main effects of magnitude of deviance, stimulus type, and ROI (*BF*_10_=3.275e +40).

There were no significant effects or interactions for the N1 although the data provide some positive evidence for the Bayesian model only for the main effect of laterality (*BF*_10_=15.961, *F*(2,40)=1.261, *p*=.294, η**^2^**=.037, ε**=**.928).

- 1. **Repetition suppression**

Although the N1 results are similar to the P2 results for the LPO and MPO regressions, the RPO’s two regression lines both showed decreasing N1 scores of essentially identical slope. We must confess we have no idea why this weird result happened (data in S5_Data).

**Reference**

[1] Smout CA, Tang MF, Garrido MI, Mattingley JB. Attention promotes the neural encoding of prediction errors. PLOS Biology. 2019;17(2):1-22. doi: 10.1371/journal.pbio.2006812.
